# The Effect of Music Intervention on Dental Anxiety During Dental Extraction Procedure

**DOI:** 10.2174/1874210601711010565

**Published:** 2017-10-31

**Authors:** Tantry Maulina, Nina Djustiana, M. Nurhalim Shahib

**Affiliations:** 1 Oral Surgery Department, Faculty of Dentistry, Universitas Padjadjaran, Bandung, Indonesia; 2 Dental Materials Science and Technology Department, Faculty of Dentistry, Universitas Padjadjaran, Bandung, Indonesia; 3 Biochemistry and Molecular Biology Department, Faculty of Medicine, Universitas Padjadjaran, Bandung, Indonesia

**Keywords:** Dental extraction, Dental anxiety, Music intervention, Noradrenaline plasma, Cortisol, Cathecolamine

## Abstract

**Background:**

In order to minimize the possibility of unsuccessful dental extraction procedure due to dental anxiety, there are several approaches that can be used, including music intervention.

**Objective:**

The objective of this research was to investigate the effectiveness of classical and religious Islamic music on reducing dental anxiety.

**Materials and methods:**

Two hundred and twenty-five muslim participants (105 males, 120 females) were recruited for this study and randomly assigned to three groups: classical music group, religious Islamic music group, and the group with no music intervention, equally in numbers. Participant’s blood pressure (BP) and blood sample were taken prior to and after dental extraction to evaluate systolic and diastolic BP as well as nor-adrenaline plasma (NAP) level. All data were then analyzed by using t-test, ANOVA test, Mann-Whitney and Kruskawallis test.

**Results:**

There was a decrease in NAP level in the religious music group (0.110 ng/mL) and the control group (0.013 ng/mL) when initial NAP level was compared to post extraction NAP level, whilst the classical music group showed an increase of 0.053 ng/mL. There were significant differences found between the religious Islamic music group and the classical music group (*p* = 0.041) as well as the control group (*p* = 0.028) for the difference between pre and post NAP level, of which the NAP level of the religious Islamic group participants were lower.

**Conclusion:**

Religious Islamic music was proven to be effective in reducing dental anxiety in Muslim participants compared to classical music. Despite, further evaluation in a more heterogenous population with various religious and cultural background is needed.

## 1 INTRODUCTION

Dental extraction has been identified as one of dental treatments that has the potential to induce dental anxiety. This might be possible due to the administration of local anaesthesia which involved the usage of a syringe. A study about dental anxiety prior to surgical tooth extraction which involved 164 Oral Surgical Patients in Pacific Dental College, India showed that 35.5% of these patients experienced fear of injection [1] which is one of the factor that can lead to the development of dental anxiety. Dental anxiety that might arise during dental extraction procedure can complicate the procedure as well as minimizing the possibilities of having a successful procedure. As patients with dental anxiety are most likely to avoid dental procedures [2-4].

Dental anxiety can be defined as a state where an individual is evoked and prepared for something to happen, with a non-specific feeling of apprehension, associated with abnormal conditions [5]. The etiology of dental anxiety can be attributed to traumatic or painful dental experiences as well as fearful attitudes learnt from dentally anxious family member [6]. Based on the origin and main sources of fear, dental anxiety patients can be categorized into four groups: the ones that are anxious about a spesific stimuli, the ones that are distrustful of dental clinicians, the ones that are generally anxious about most things, and the ones that are frighten that medical emergencies might occur during their dental treatment [7].

Regardless of the classification of dental anxiety, patient with dental anxiety will show some refusal symptoms during treatment. These refusal symptoms of anxiety, can be classified into physiological symptoms, behavioural symptoms, cognitive symptoms, and emotional symptoms [8]. Among these symptoms, the physiological symptoms, such as dyspnea, hyperventilation, tachycardi, hypertension, increased respiration rate, nausea, and vomiting, are the most potential symptoms that might attribute to the failure of the treatment [8]. These symptoms are the manifestation of the secretion of stress hormones such as cortisol, and cathecolamine (noradrenaline and adrenaline) [9]. Whilst cortisol is known as the stress hormone that is released as a response to anxiety in a long term, cathecolamines are the stress hormones that are released during short term anxiety [10]. As the anxiety during dental treatment falls into a category of a short term anxiety, the measurement of noradrenaline plasma level can be used as dental anxiety indicator.

In order to minimize the potentials of these symptoms to occur and failing dental procedure, dental practitioners have been trying to apply intervention methods that are expected to reduce dental anxiety level, namely hypnodontic, sedation, and sound therapy. The usage of sound or music as a theurapeutic tool has been known for quite sometime. One of the most used music genres in the medicine field is classical music. Due to its calming and soothing tempo, classical music has been well known for its efficacy in reducing anxiety [11-13]. Aside from classical music, another type of sound or music that has been used to decrease anxiety as well as depression is religious Islamic music or the rythmic Quranic recitation [14, 15].

Despite of the study results that revealed the effectiveness of music in reducing anxiety [16, 17], there were study results that revealed just the opposite [18, 19]. Therefore, based on these contradictive previous study results, the aim of this study was to investigate the effectiveness of classical music compared to religious Islamic Music in decreasing dental anxiety during dental extraction. The present study used systolic and diastolic blood pressure measurement as well as nordrenaline plasma level as anxiety indicators and analyzed these indicators statistically in order to reveal the expected reduction in dental anxiety.

## 2 METHODOLOGY

Two hundred and twenty-five participants (105 male, 120 females; aged 18-45 years old) who came to the Exodontia Clinic of Faculty of Dentistry, Universitas Padjadjaran, Bandung, Indonesia to have dental extraction, were recruited for this study. Prior to the start of the study, an ethical clearance was obtained from the Profession and Research Ethics Committee of Medical Committee Faculty of Dentistry Dental Hospital Padjadjaran University (ethical clearance number:62/H6.28/RSGM/PL/2016). As required, procedures and ethical aspect of the current research has been conducted in full accordance with the World Medical Association Declaration of Helsinki and that all participants gave their written consent for their participation in the current study and the usage of any of their photograph(s), that was taken during the study that was related to the study procedure, in all possible publication related to the study.

Recruited participants were divided into three groups randomly. The first group was the control group, where no music intervention was given; the second group was the classical group, where the participants listened to classical music during tooth extraction procedure; and the third group was the Islamic music group, the participants of which listened to Islamic music during tooth extraction. Each group consisted of 75 participants that were randomly assigned by the field researcher (TM). Prior to the experiment, all participants were asked to fill out the Modified-Modified Dental Anxiety Scale (MDAS) (Apendix 1). This particular questionnaire is a modification from the Modified Dental Anxiety Scale (M-MDAS). The questions in the questionnaire have been modified to address dental anxiety that is specifically related to tooth extraction procedure. The questionnaire has been validated prior to the usage in this study.

The M-MDAS questionnaire was used as a filtering tool in order to objectively screened anxious participants. Only participants with a score between 10-18 can be included in this study. Ten was chosen as the baseline score, which meant that the participant is classified as anxious (slightly anxious), whilst 18 was chosen because it is the maximum score for someone to be classified as fairly anxious. Those with a score of 19 or beyond were considered as highly anxious (phobic) dental patients and therefore, were excluded from this study. Another inclusion criterions for participants were: ages between 18-45 years old, had an upper first molar teeth that satisfies the extraction criterion, never had tooth extraction prior to the current one, had a systolic blood pressure within the range of 110-120 mmHg and diastolic blood pressure within the 70-80 mmHg range, and willing to participate in this study. At the time of experiment, all participants were Muslim participants. There were no participants from another religious beliefs as the investigation was focused on an intra belief effect before investigating the effect on two different beliefs. As for participants who were pregnant, presented cardiovasculair abnormalities, had a psychological-anxiety related disorder, illiterate, and had listening abnormalities were excluded from the study. Eligible participants were then informed about the procedure, and once the participant signed the informed consent, the field researcher took a closed envelope that contained the name of the group of which the participant were allocated to.

There were two types of music that were used in this study. The first one was classical music, and the second one was religious Islamic music. Both types had 60-70 beats per minute with a frequency between 750 – 3000 Hz. The tittle of the classical music used was Molto Allegro by Mozart, whilst the religious Islamic music used were songs sang by Malaysian vocal group named Raihan. All participants listened to the music through an ear phone that was connected to a digital music player. In order to avoid any bias, the extraction operator was not informed about the type of music listened by the participant. After participant were seated, the first blood pressure measurement were taken by using a digital blood pressure monitor from Omron (OMRON HEM-8172, JAPAN). After blood presure measurement was completed, a trained nurse then took the first blood sample from the fossa cubitus as much as 2 ml for noradrenaline plasma level measurement. Noadrenaline plasma (NAP) level was measured by using Enzyme-linked immunosorbent assay (ELISA) technique.

For those who were allocated to the first group (control group), the next procedure was the act of local anaesthesia, followed by the dental extraction procedure. Whilst for those who were in the second and third group, the next step was to listen to the music. Five minutes after the music was listened by the participant, the act of local anaesthesia was performed followed by the extraction procedure. All local anesthesia injection as well as tooth extraction was performed by one operator. After the extraction procedure was completed, all participants from all groups were assigned to the second blood pressure measurement. Another blood sample was then drawn for the second time by the same nurse. The blood sample were then analyzed by using the ELISA technique to reveal the value of the nor-adrenaline plasma level. The data gained from this study were analyzed by using t-Test and Analysis of Variance (ANOVA) if the data are normally distributed. If the data are not normally distributed, the tests used were the Mann-Whitney test and the Kruskawallis test. As all participants were eligible and completed the study, all data were able to be gained and analyzed by the previously mentioned statistical analysis. All statistical analysis was performed by using SPSS version 22 (IBM, USA).

## 3 RESULTS

The participants of this study consist of 225 participants who were at least 18 years of age. The youngest participant was in the religious Islamic group whilst the eldest is in the classical music group. Age is one of the factor that is highly considered in this study considering that dental anxiety tends to decrease along with increased age [20]. The demographical characteristics that consists of age, gender, and educational attainment of the participants is listed in Table **1** whilst the total mean score for M-MDAS score for each group are illustrated in Table **2**.

**Fig. (1):**
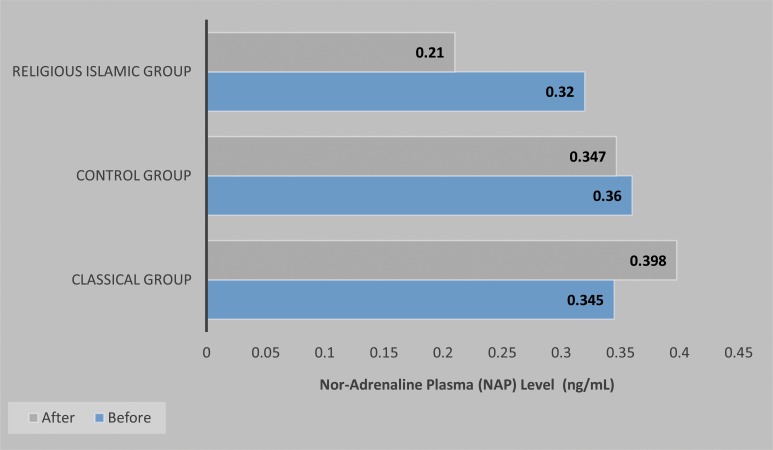
The pre and post-extraction comparison of the noradrenalin plasma (NAP) level between Classical music group, Religious Islamic music group, and Control Group.

An evaluation of the NAP level consisted of a comparison between the NAP level prior to the extraction and the NAP level after the extraction for all groups (Fig. **1**). The NAP level in classical group, showed an increase of 0.053 ng/mL, whilst the NAP level of the participants in the religious Islamic group showed a decrease of 0.110 ng/mL, and the NAP level of the control group showed a decrease of 0.013 ng/mL. A statistical analysis was then performed on these data. Since the data were normally distributed, an ANOVA test was performed to test the difference between post extraction NAP level and the initial NAP level between each group, whilst the Kruskalwallis test was performed on the post-extraction NAP level between each group. There was a significant difference between the classical music group and the religious Islamic music group (*p* = 0.041) as well as between religious Islamic group and control group (*p* = 0.028) for the difference between pre and post NAP level as well as the post-extraction noradrenaline plasma value (*p*=0.032).

As for the systolic and diastolic blood pressure, a comparison of systolic blood pressure and diastolic blood pressure, between each group prior and after the extraction procedure is illustrated in Table **3**. A statistical analysis by using the ANOVA test on systolic blood pressure value revealed that there were no significant difference between each group on initial systolic blood pressure level (*p*=0.63), as well as post-extraction systolic blood pressure level (*p*=0.57). There was a significant difference found for systolic blood pressure between the classical music group and the religious Islamic music group (*p* = 0.037) as well as between religious Islamic group and control group (*p* = 0.023) for the difference between pre and post systolic blood pressure. An ANOVA test result on initial diastolic blood pressure between groups revealed no significant difference for any variables compared.

## 4 DISCUSSION

There are several mechanism that underlies dental anxiety, one of which, an increase of the sympathetic and parasympathetic nerve system activities [21]. An increased activity of the symphatetic nerves will then be followed by an increase in noradrenaline secretion and therefore causing an increase in blood pressure, heart rate, and muscle contractility [22-24]. Part of the results of the present study was in-line with the above theory (Table **3**). The allocation of music is supposed to activate the parasympathetic nerve system and hence inducing the parasympathetic system to be more dominant than the sympathetic system. This mechanism will affect the decrease of the muscle contractility as well as the heart rate [22, 25]. Therefore in general music is expected to reduce anxiety, including dental anxiety [18, 26].

In the present study, participants who listened to classical music tend to show an increase in the NAP level, the systolic blood pressure, as well as the diastolic blood pressure. This particular results of the study is different from most previous studies [27, 28] that showed the decrease of blood pressure during music intervention. Despite of the difference from the results of previous studies, there is one study conducted by Chafin *et al*. that was partially inline with the result of the present study. In their study, Chafin **et al**. stated that different type of music might have different effect on blood pressure [29]. This particular result of the study might be due to the fact that the participants of the current study were all Indonesian and were not familiar with classical music. Classical music might be a familiar tone for those originated from European countries, yet, it might not be the case for Indonesian people. A study by Mok and Wong (2003) that evaluated the effect of music on patient’s anxiety showed that participants who listened to the music they prefered will show more significant decrease [30].

Another study by Aitken **et al**. [31] about the efficacy of music therapy on forty-five children aged 4-6 years old who were about to undergo dental treatment showed no significant differences statistically between those who listened to upbeat music group, relaxing group, and no music group. As for the increase of the diastolic blood pressure, it might be due to the fact that the increase of the NAP plasma level during anxiety will cause vasocontriction of blood vessels in general, which will cause total perifer resistance [22]. An increase in total perifer resistance will increase the diastolic blood pressure [32, 33].

Unlike the participants of the classical music group, the participants who listened to religious Islamic music showed a decrease in systolic blood pressure, diastolic blood pressure, as well as NAP level. This part of the present study showed that music reduced dental anxiety as expected and was inline with a study conducted by Sevban *et al*. about the effect of music on anxiety in patients that were about to undergo surgery. In that study, it was revealed that the level of anxiety prior to surgery was significantly reduced when patient were exposed to music intervention [34]. Another study by Bradshaw *et al*. about the effect of religious music on anxiety experienced by the elderly showed that religious music was proven to be effective in reducing anxiety [35].

Another mechanism that might be responsible for the reduction of the blood pressure and NAP level in the Islamic music group is the familiarity of the Islamic music used in the current study. As a country with the biggest Muslim population, Islamic music is something familiar to Indonesian. A study by Sung **et al**. about the effect of familiar music intervention on anxiety experienced by the elderly suggested that the familiarity of the music might recall certain pleasant memories associated with the music and that it might elicit the patient’s positive feeling and therefore is responsible for the reduced anxiety [36].

## CONCLUSION

Religious Islamic music intervention for Indonesian muslim patients who were about to undergo dental extraction procedure was proven to be effective in decreasing systolic blood pressure, diastolic blood pressure, and nordrenaline plasma level, whilst classical music did not seem to be as effective in this particular group of patients. In regards to the current results, considering the fact that all participants were muslims with a homogenous cultural background, further study investigating the effect of religious Islamic music on patients with more diversified religious beliefs as well as racial and cultural background will provide valuable scientific insight in this particular area of study.

## Figures and Tables

**Table 1:** Participants’ demographical distribution for each group.

	**Classical Group**	**Religious Group**	**Control Group**
**Gender**			
Male	31	39	35
Female	44	36	40
Age (Mean Value)	33.56 years old	32,34 years old	28,5 years old
Educational Attainment			
High School	57	60	65
College / University	18	15	10

**Table 2:** Modified ‒ Modified Dental Anxiety Score (M-MDAS) total mean score for each group.

**Questions**	**First Group** **(Control Group)**	**Second Group** **(Classical Music)**	**Third Group** **(Religious Islamic Music)**
Question number 1	2.85	2.75	2.70
Question number 2	2.75	2.50	2.85
Question number 3	3.50	3.10	2.90
Question number 4	3.20	3.30	3.50
Question number 5	3.70	3.55	3.45
Total mean score	16	15.20	15.40

**Table 3:** Pre and post-extraction systolic blood pressure (mm/Hg) and diastolic blood pressure (mm/Hg) comparison between groups.

	**Classical music group**			**Control group**			**Religious Islamic music group**		
	Pre	Post	Diff*	Pre	Post	Diff*	Pre	Post	Diff*
**Systolic Blood Pressure**	129.77	132.23	↑2.46	136.76	135.55	↓1.21	126.55	120.32	↓6.23
**Diastolic Blood Pressure**	79.20	81.46	↑2.26	80.85	79.32	↓1.53	80.34	78.52	↓1.82

*Differences

## LIST OF ABBREVIATIONS

BP: Blood pressure
NAP: Nor-Adrenaline Plasma
VAS: Visual analogue scale
M-MDAS: Modified-Modified dental anxiety score
ELISA: Enzyme-linked immunosorbent assays
ANOVA: Analysis of variance
