# Supplementary material for: The Effect of Music Intervention on Dental Anxiety During Dental Extraction Procedure
Source: Open Dent J. 2017 Oct 31;11:565–72. doi: 10.2174/1874210601711010565 (PMC5712637; doi:10.2174/1874210601711010565)
Supplement: Supplementary file 1 [file TODENTJ-11-565_SP.pdf]

Send Orders for Reprints to reprints@benthamscience.ae

BENTHAM OPEN

CrossMark

The Open Dentistry Journal

Supplementary Material

Content list available at: www.benthamopen.com/TODENTJ/

DOI: 10.2174/1874210601711010565

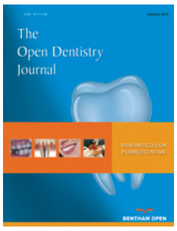

The Effect of Music Intervention on Dental Anxiety During Dental Extraction Procedure

Tantry Maulina[1,*], Nina Djustiana[2] and M. Nurhalim Shahib[3]

[1]*Oral Surgery Department, Faculty of Dentistry, Universitas Padjadjaran, Bandung, Indonesia*
[2]*Dental Material Science and Technology Department, Faculty of Dentistry, Universitas Padjadjaran, Bandung, Indonesia*
[3]*Molecular Biology Department, Faculty of Medicine, Universitas Padjadjaran, Bandung, Indonesia*

CAN YOU TELL US HOW ANXIOUS YOU GET, IF AT ALL, WITH YOUR TOOTH EXTRACTION PROCEDURE?

PLEASE INDICATE BY INSERTING 'X' IN THE APPROPRIATE BOX

Appendix 1. Modified "Modified Dental Anxiety Scale" (MDAS)

**1. If you were told that your tooth has to be extracted, how would you feel?**					
	Not Anxious □	*Slightly Anxious* □	*Fairly Anxious* □	*Very Anxious* □	*Extremely Anxious* □
**2. If you were about to go to the dentist tomorrow to have your tooth extracted, how would you feel?**					
	Not Anxious □	*Slightly Anxious* □	*Fairly Anxious* □	*Very Anxious* □	*Extremely Anxious* □
**3. If you were sitting at the dentist waiting room for your tooth extraction procedure, how would you feel?**					
	Not Anxious □	*Slightly Anxious* □	*Fairly Anxious* □	*Very Anxious* □	*Extremely Anxious* □
**4. If you were about to have a local anesthetic injection in your gum, how would you feel?**					
	Not Anxious □	*Slightly Anxious* □	*Fairly Anxious* □	*Very Anxious* □	*Extremely Anxious* □
**5. If you were about to have your wisdom tooth extracted through a simple surgery procedure, how would you feel?**					
	Not Anxious □	*Slightly* Anxious □	*Fairly* Anxious □	*Very* Anxious □	*Extremely* Anxious □

Instructions for scoring (remove this section below before copying for use with patients)

The Modified Dental Anxiety Scale. Each item scored as follows:

Not anxious = 1

Slightly anxious = 2

Fairly anxious = 3

Very anxious = 4

Extremely anxious = 5

Total score is a sum of all five items, range 5 to 25: Cut off is 19 or above which indicates a highly dentally anxious patient, possibly dentally phobic
